# Site-dependent and interindividual variations in Denonvilliers’ fascia: a histological study using donated elderly male cadavers

**DOI:** 10.1186/s12894-015-0034-5

**Published:** 2015-05-12

**Authors:** Kuniyasu Muraoka, Nobuyuki Hinata, Shuichi Morizane, Masashi Honda, Takehiro Sejima, Gen Murakami, Ashutosh K Tewari, Atsushi Takenaka

**Affiliations:** Division of Urology, Department of Surgery, Tottori University Faculty of Medicine, Yonago, 683-8504 Japan; Department of Urology, Kobe University Graduate School of Medicine, Kobe, Japan; Division of Internal Medicine, Iwamizawa Kojin-kai Hospital, Iwamizawa, Japan; Department of Urology, Mount Sinai Hospital, New York, NY USA

**Keywords:** Denonvilliers’ fascia, Fascia propria of the rectum, Neurovascular bundle, Prostatic capsule, Prostate, Robot-assisted radical prostatectomy

## Abstract

**Background:**

Site-dependent and interindividual histological differences in Denonvilliers’ fascia (DF) are not well understood. This study aimed to examine site-dependent and interindividual differences in DF and to determine whether changes in the current approach to radical prostatectomy are warranted in light of these histological findings.

**Methods:**

Twenty-five donated male cadavers (age range, 72–95 years) were examined. These cadavers had been donated to Sapporo Medical University for research and education on human anatomy. Their use for research was approved by the university ethics committee. Horizontal sections (15 cadavers) or sagittal sections (10 cadavers) were prepared at intervals of 2–5 mm for hematoxylin and eosin staining. Elastic–Masson staining and immunohistochemical staining were also performed, using mouse monoclonal anti-human alpha-smooth muscle actin to stain connective tissues and mouse monoclonal anti-human S100 protein to stain nerves.

**Results:**

We observed that DF consisted of disorderly, loose connective tissue and structures resembling “leaves”, which were interlacing and adjacent to each other, actually representing elastic or smooth muscle fibers. Variations in DF were observed in the following: 1) configuration of multiple leaves, including clear, unclear, or fragmented behind the body and tips of the seminal vesicles, depending on the site; 2) connection with the lateral pelvic fascia at the posterolateral angle of the prostate posterior to the neurovascular bundles, being clear, unclear, or absent; 3) all or most leaves of DF fused with the prostatic capsule near the base of the seminal vesicles, and periprostatic nerves were embedded in the leaves at the fusion site; and 4) some DF leaves fused with the prostatic capsule anteriorly and/or the fascia propria of the rectum posteriorly.

**Conclusions:**

Site-dependent and interindividual variations in DF were observed in donated elderly male cadavers. All or most DF leaves are fused with the prostatic capsule near the base of the seminal vesicles and some DF leaves are fused with the fascia propria of the rectum posterior. Based on our results, surgeons should be aware of variations and search for them to create a suitable dissection plane to avoid iatrogenic positive margins and rectal injury.

## Background

Since Charles-Pierre Denonvilliers first discovered the firm, membranous structure between the rectum and prostate or bladder in 1836, now called “Denonvilliers’ fascia” (DF), the origin of DF has remained controversial. The most widely accepted theory is that DF represents fusion of the embryonic peritoneum of the retrovesical cul-de-sac [1,2]. Disorderly, loose connective tissue is present between the cul-de-sac and the rectourethralis muscle (RUM), with a tight, thick membrane that includes smooth muscle fibers between the cul-de-sac and the posterior aspect of the prostate near the base of the seminal vesicle [3]. DF is often fused with the prostatic capsule at the center of the posterior prostatic surface [4-6]. DF is not well adhered to the prostatic capsule toward the posterolateral aspect of the prostate. The space between DF and this capsule is filled by areolar tissue and the neurovascular bundle (NVB) [5-7]. Costello et al. [8] demonstrated plexus- or mesh-like nerves extending along the posterior aspect of the prostate after removal of DF using cadaveric dissection. Laterally, DF becomes continuous with the “pararectal fascia” posteriorly and the “lateral pelvic fascia” (LPF) anteriorly.

DF has been used as a surgical landmark during intrapelvic surgery for many years, and is one of the major areas of interest for urological surgeons for cancer control and functional preservation. Since the operative procedure for robot-assisted radical prostatectomy (RARP) was established by Menon et al. [9], it has widely expanded is use. RARP provides a precise, highly magnified view and less venous oozing, leading to a better anatomical understanding of fine membranous structures.

To the best of our knowledge, site-dependent and interindividual histological differences in DF have not previously been investigated. Therefore, this study aimed to examine site-dependent and interindividual differences in DF among 25 donated male cadavers and to determine whether changes in the current approach to radical prostatectomy are warranted in light of these histological findings. In the present study, DF was defined as a structure between the rectum and prostate, bladder, or seminal vesicles.

## Methods

Twenty-five donated male cadavers (mean age at death, 85 years; range, 72–95 years) were examined. None of the patients had undergone abdominal surgery based on the medical records and macroscopic observations after opening the abdominopelvic cavity. These cadavers had been donated to Sapporo Medical University for research and education on human anatomy. Their use for research was approved by Sapporo Medical University ethics committee and all the participants provided written informed consent in their lifetime. Each donated cadaver had been fixed by arterial perfusion of 10% v/v formalin solution and stored in 50% v/v ethanol solution for more than 3 months. From each cadaver, one large tissue block including the bladder neck, seminal vesicles, membranous urethra, prostate, and rectal anterior wall, as well as connective tissue around these viscera, was obtained. After trimming the tissue block, routine procedures for paraffin-embedded histological examination were performed. Horizontal sections (15 cadavers) or sagittal sections (10 cadavers) were prepared at 2- to 5-mm intervals for hematoxylin and eosin staining (HE) and immunohistochemistry. When the prostate was large, the superior and inferior halves were included in two separate paraffin blocks. From each cadaver, 20–80 HE-stained and unstained sections were prepared.

Based on observations of HE-stained sections, some sections were used for immunohistochemistry and elastic–Masson staining (a variation of Masson–Goldner staining) [10,11]. The primary antibodies that were used to identify connective tissues and nerves were mouse monoclonal anti-human alpha-smooth muscle actin (1:100, Dako M0851; Dako, Glostrup, Denmark) and mouse monoclonal anti-human S100 protein (1:200 dilution, Dako Z0311; Dako), respectively. The smooth muscle antibody yields positive results not only for any smooth muscle, but also for vascular endothelium [12].

The study was performed in accordance with the provisions of the Declaration of Helsinki 1995 (as revised in Edinburgh 2000).

## Results

### Basic structure of DF

DF was composed of collagen, elastic, and smooth muscle fibers in all cases. There were structures resembling “leaves”, actually representing elastic or smooth muscle fibers, in DF. DF consisted of a configuration of multiple leaves, mainly anteriorly, and a disorderly loose connective tissue, mainly posteriorly. Furthermore, although the multiple-leaf configuration appeared to be a firm membranous structure, it was actually recognized as a fascicle of multiple leaves with interlacing branches.

### Findings of DF from the seminal vesicle to the middle of the prostate in horizontal sections

**Figure 1:**
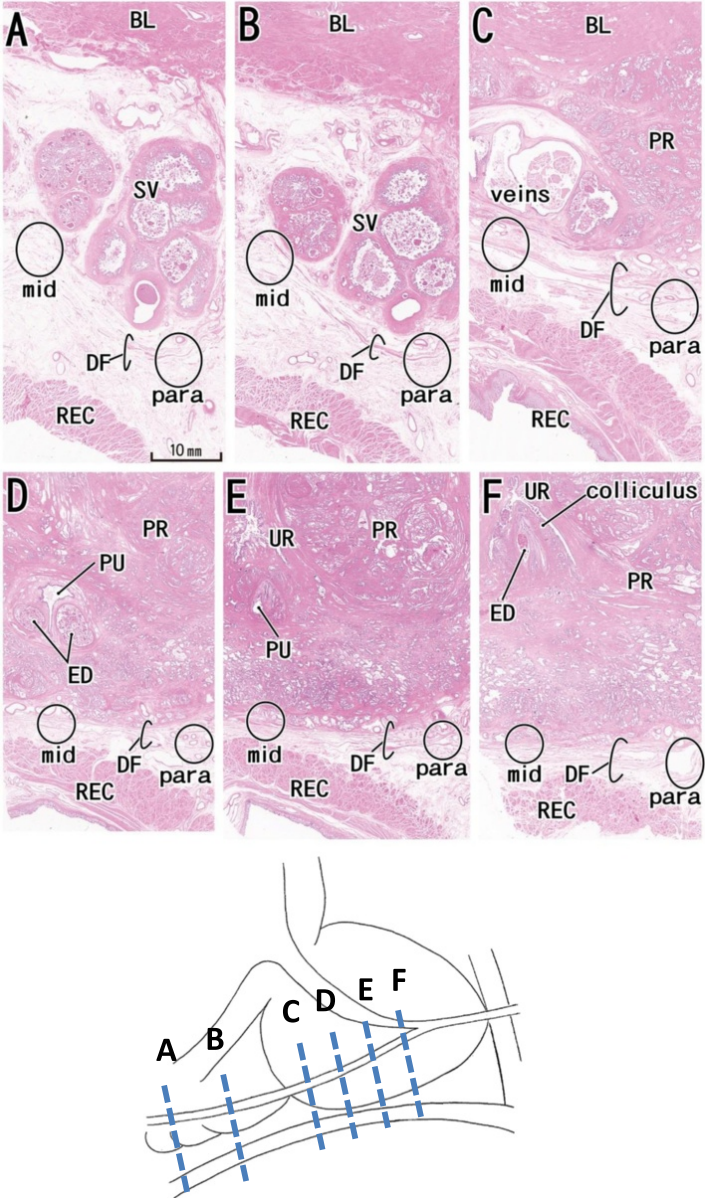
Site-dependent difference in Denonvilliers’ fascia in horizontal sections from a 90-year-old man. Panel **A** **(F)** displays the most superior (inferior) level in the figure. Intervals between panels are 3 mm **(A–B)**, 13 mm **(B–C)**, 11 mm **(C–D, D–E)**, and 6 mm **(E–F)**. Denonvilliers’ fascia (DF) shows a multiple-leaf configuration at all levels and sites. In the midsagittal areas (circle with “mid”), the fascia is unclear or fragmented in panels **A**, **B**, and **D**, while in the parasagittal areas (circle with “para”), DF is most evident in panels **B**, **C**, and **E**. The fascia thus appears more clearly identifiable in the lateral sites than in medial sites. The site more lateral to this figure is shown in Figure **2** **C** (near panel **B**) and Figure **2** **D** (near panel **E**). All panels were prepared at the same magnification (scale bar is in panel **A**). BL; bladder; ED: ejaculatory duct; PR: prostate; PU: prostatic utricle; REC: rectum; SV: seminal vesicle; UR: urethra.

**Figure 2:**
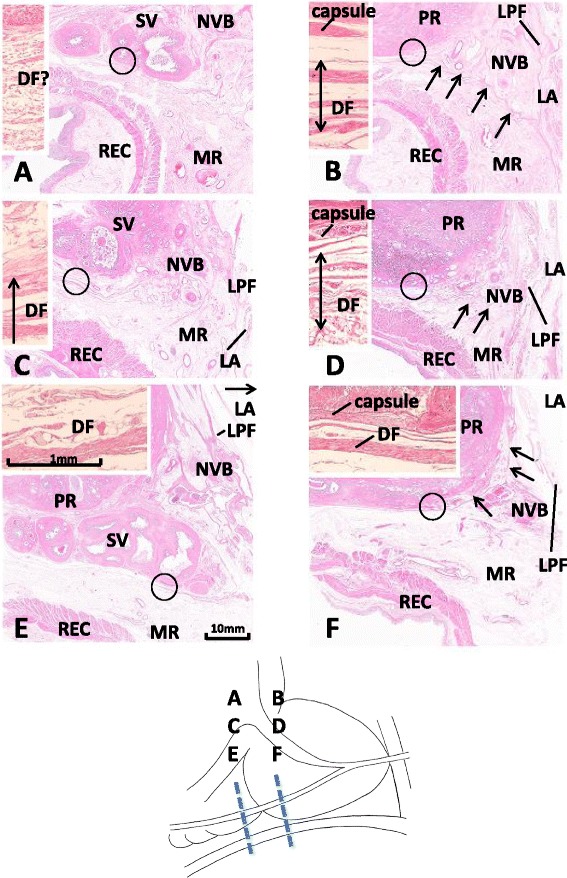
Connection of Denonvilliers’ fascia with the lateral pelvic fascia in horizontal sections. Panels **A** and **B** show an 86-year-old man. Panels **C** and **D** show a 90-year-old man. Panels **E** and **F** show an 83-year-old man. The right- and left-hand side columns display Denonvilliers’ fascia (DF) behind the base of the seminal vesicle (SV) and prostate (PR), respectively. The insert of each panel shows a higher magnification view of a circle in the panel. In panel **B**, DF (arrows) extends laterally in the posterior side of the neurovascular bundle (NVB) to connect with the lateral pelvic fascia (LPF). In panel **D**, the fascia can be seen (arrows), but the connection is not clear. In panel **F**, the fascia does not extend laterally, but extends anteriorly (arrows) instead along the prostatic capsule. All panels (or all inserts) were prepared at the same magnification (scale bars are in panel **E** and its insert). In the six inserts, DF shows a multiple-leaf configuration in the first and second specimens (86- and 90-year-old men), while the fascia is composed of a thick leaf and other thin leaves in the third specimen (83-year-old man). The multiple-leaf configuration is unclear in the insert of panel **A**. LA: levator ani muscle; LPF: lateral pelvic fascia; MR: mesorectum; REC: rectum.

In contrast to sagittal sections, horizontal sections showed the presence of multiple leaves of DF, and two to eight leaves were consistently observed (Figures 1 and 2). Because of the multiple leaves, we found it difficult to determine whether the leaves were “thick or thin”. However, when we compared these sections with those from a specimen from a 90-year-old man, DF leaves tended to be more clearly observed behind the middle of the seminal vesicles (Figure 1B) and near the base of the seminal vesicles (Figure 1C). In contrast, in sites behind the superior half of the seminal vesicles, DF leaves tended to be unclear or fragmented (Figure 1A) in the midsagittal areas.

When individual variations in DF were investigated, clear variation was observed in DF morphology behind the seminal vesicles and at the posterolateral angle of the prostate. With regard to the multiple-leaf configuration of DF behind the base of the seminal vesicles, some were clear (Figure 2C, E), and some were unclear (Figure 2A). DF leaves extended to the posterior side of the NVB to connect with the LPF in seven cadavers (Figure 2B), but the connection was unclear (Figure 2D) or absent (Figure 2F) in eight cadavers. In the seven cadavers in which a lateral fascial connection was evident on the posterolateral side of the prostate, DF usually became unclear on the posterolateral side of the seminal vesicles. In the eight cadavers with an unclear or absent connection, DF extended anteriorly to pass through or disperse in the NVB, and some leaves joined the capsule covering the lateral aspect of the prostate. The mesorectal loose tissue thus appeared to be continuous with the NVB (Figure 2F). Among multiple leaves, a single leaf was sometimes thick at a limited area or level (Figure 2E, F), although quantitative evaluation was difficult because of the use of semiserial sections for observations.

### Findings of DF from the middle of the prostate to the apex in sagittal sections

**Figure 3:**
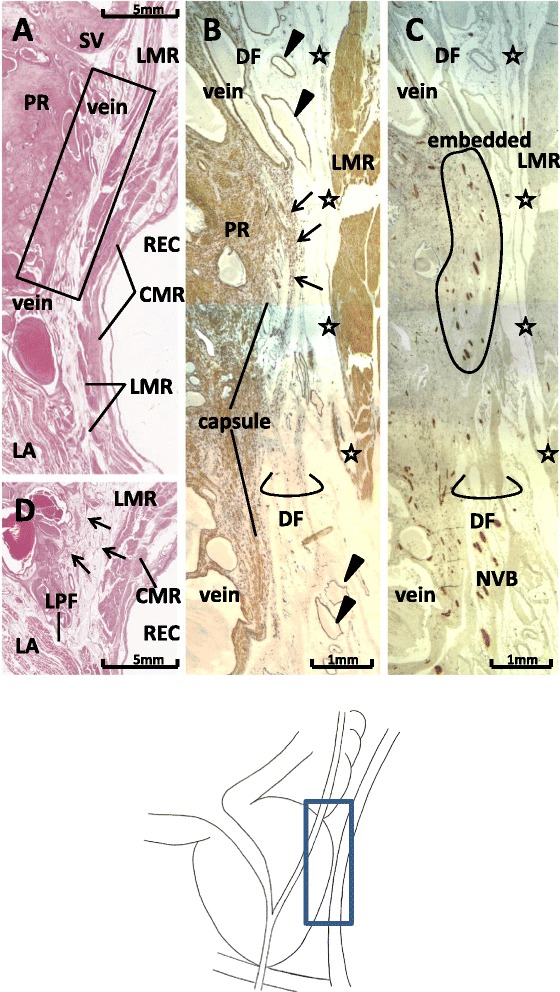
Fascial complex between Denonvilliers’ fascia and the prostatic capsule in sagittal sections. Sections from a 75-year-old man are shown. Panel **A** (HE staining) displays topographical anatomy around Denonvilliers’ fascia (DF) at the 2- to 3-o’clock position of the rectum. The levator ani muscle (LA) approaches the rectum (REC). Panel **B** (Panel **C**), corresponding to a square in panel **A**, shows immunohistochemistry for smooth muscles (for all nerves). DF, containing smooth muscles, comprises 3–4 leaves (lower part of panel **B**), but the leaves are bundled to fuse with the prostatic capsule (capsule) in the upper part of the panel (arrows). At the fusion area (panel **C**), periprostatic nerves are embedded in the fascial complex between DF and the prostatic capsule (encircled). Stars indicate a candidate for the fascia propria of the rectum. The DAKO antibody that was applied for smooth muscles also strongly stains vascular endothelium (arrowheads). In panel **D**, 4 mm lateral to the area shown in panel **A**, fascial leaves (arrows) become thinner and fewer in number. CMR and LMR: circular and longitudinal muscle layers of the rectum; LP: lateral pelvic fascia; NVB: neurovascular bundle at the posterolateral corner of the prostate; PR: prostate; SV: seminal vesicle.

**Figure 4:**
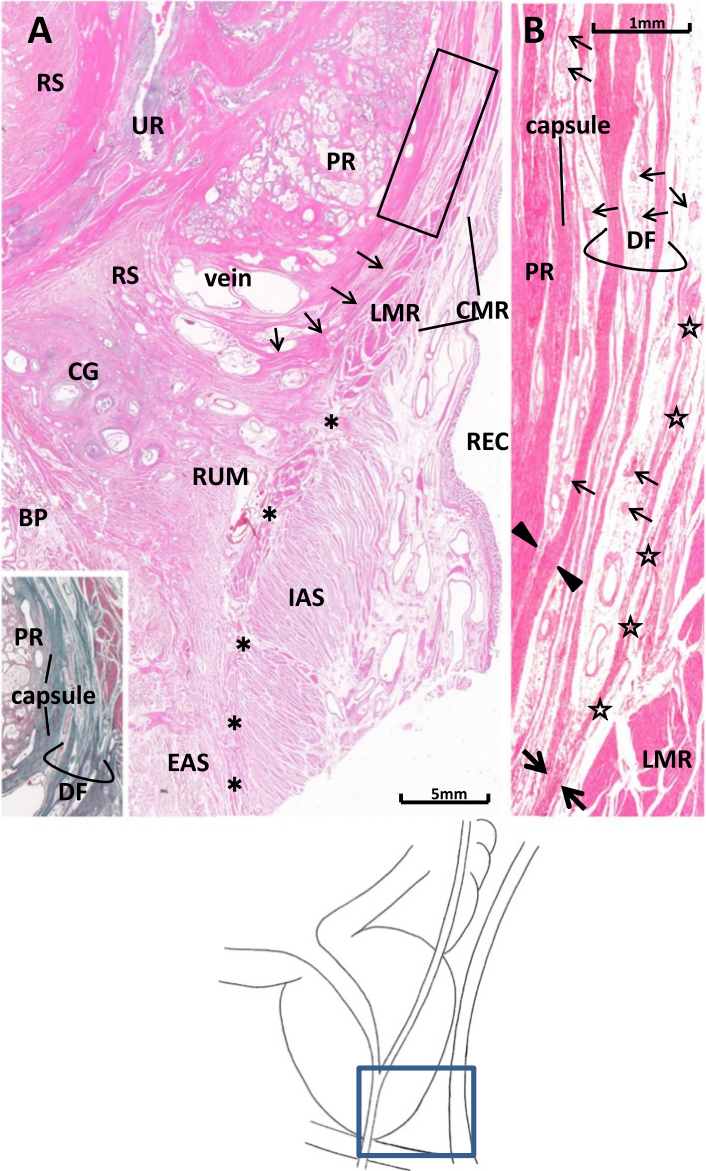
Multiple-leaf configuration of Denonvilliers’ fascia in a midsagittal section from a 79-year-old man. Panel **A** displays topographical anatomy around Denonvilliers’ fascia (DF). The longitudinal muscle layer of the rectum (LMR) extends inferiorly to continue to the conjoint muscle coat or longitudinal anal muscle (asterisks). The leaves of DF (arrow) are dispersed in a venous plexus (veins) without ending at the rectourethralis muscle (RUM) in this specimen. The insert in the panel **A** (elastic–Masson staining; a section near panel **A**) shows rich content of elastic fibers (black color) in the prostatic capsule, DF, and fascia propria of the rectum. Panel **B**, corresponding to the rectangle in panel **A**, shows the multiple-leaf configuration of DF. Periprostatic nerves (thin arrows) are scattered between the fascial leaves. A thick leaf of the fascia is fused with the prostatic capsule (capsule) at the site indicated by arrowheads, while the other leaf merges with the fascia propria of the rectum (stars) at a site indicated by thick arrows. BP: bulbus penis; CG: Cowper’s gland, CMR: circular muscle layer of the rectum; EAS: external anal sphincter; IAS: internal anal sphincter; PR: prostate; REC: rectum; RS: rhabdosphincter area; UR: urethra.

Even when a monolayer configuration appeared in one or several sections, this was restricted to a small area, and DF actually comprised multiple leaves and loose connective tissue (Figures 3 and 4). Notably, when the fascial space between the prostate and rectum was narrower than 3 mm without interposition of the mesorectal loose tissues at or near the midsagittal line, all or most DF leaves were bundled to fuse with the prostatic capsule (Figure 3A, B). The fusion site was equivalent near the base of the seminal vesicles.

Periprostatic nerves ran between multiple leaves, and all or most periprostatic nerves appeared embedded in the fascial complex between DF leaves and the prostatic capsule (i.e., at the fusion site) (Figure 3C). These embedded nerves were not observed in all areas behind the prostate, but were mostly restricted within a region associated with DF middline adherence to the capsule of 10 mm wide × 5 mm high at maximum.

Elastic fibers were rich throughout the entire capsule, the fascia propria of the rectum, and DF (Figure 4A, insert). In the multiple leaves of DF, some leaves were fused with the prostatic capsule (Figures 3B and 4B) and/or the fascia propria of the rectum (Figure 4B). The latter connection was particularly evident in sagittal sections, because the fascia propria of the rectum became evident along the external longitudinal smooth muscles of the rectum.

Leaves of DF were dispersed around the venous plexus on the superior side of the RUM (Figures 3A and 4A), while parts of the longitudinal muscle layer of the rectum were dispersed into the latter smooth muscle mass. At least three specimens showed parts of the DF ending at the RUM (figure not shown). Inferiorly, DF reached a level behind the rhabdosphincter area, but the venous plexus consistently interposed between the striated muscle and the inferior end of DF (Figure 4A). The connection of DF with the LPF was difficult to observe in sagittal sections because of the venous plexus interposed in the lateral site, including the levator ani muscle (Figure 3).

## Discussion

The present study showed that the configuration of the firm membranous structure of DF consistently involved multiple leaves. Even when a monolayer configuration appeared, this was restricted to a small area and actually comprised a thick leaf and other thin leaves. As Bertrand et al. [13] suggested, DF variations appeared to depend on differences in mechanical stress from either or both the prostate and rectum. Site-dependent and interindividual variations in DF were also observed, including the following: 1) variations in configuration behind the seminal vesicles; 2) variations in the connection with the LPF at the posterolateral angle of the prostate; 3) fusion of all or most DF leaves with the prostatic capsule near the base of the seminal vesicles; and 4) fusion of some DF leaves with the prostatic capsule anteriorly and/or the fascia propria of the rectum posteriorly.

**Figure 5:**
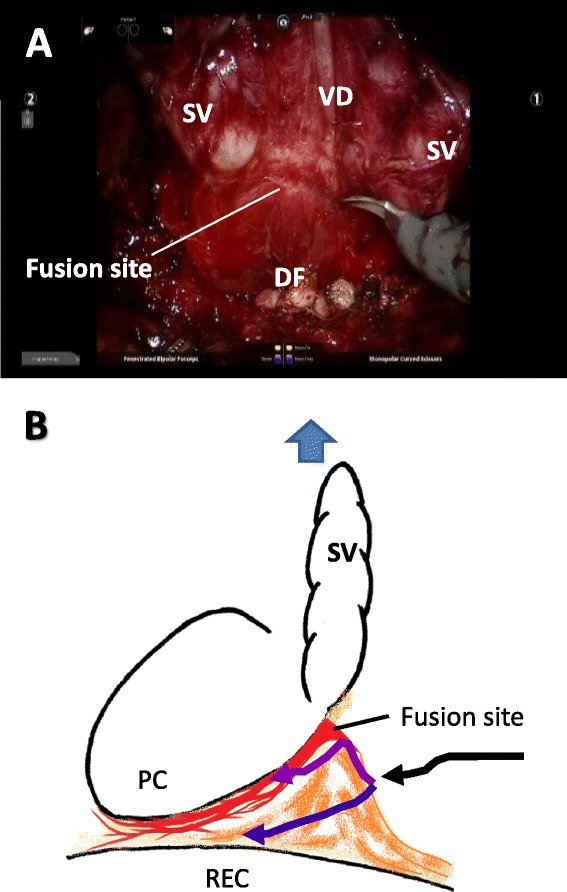
Intraoperative findings **(A)** and schema **(B)** of Denonvilliers’ fascia. When the seminal vesicles and vas deferens are pulled ventrally, Denonvilliers’ fascia (DF) is observed as a membranous structure, and the fusion site of DF with the prostatic capsule is recognized near the seminal vesicle-prostate junction (Panel **A**). DF between the seminal vesicles and rectum should first be cut at the midline to avoid entering the prostatic capsule (black arrow). After this incision, a mesh-like structure is found behind the posterior aspect of the prostate. With a nerve-sparing procedure, the dissection plane should be as close to the prostate as possible (purple arrow). If there is advanced cancer at the border of the posterior aspect of the prostate, the dissection plane can remain adjusted to the rectal wall (blue arrow). PC: prostatic capsule; REC: rectum; SV: seminal vesicle; VD: vas deferens.

Between the seminal vesicles and rectum, some DF leaves were clearly evident, while others were unclear or fragmented. During RARP, after bladder neck transection and dissection of the seminal vesicles and vas deferens, the connective tissue is present in the dorsum of the seminal vesicles and vas deferens. When the seminal vesicles, vas deferens, and prostate are pulled ventrally, DF is recognized as a membranous structure between the prostate and the rectum by its tension, irrespective of whether the leaves of the DF are clear or unclear histologically (Figure 5A).

Clear variation in DF morphology was also observed in the connection with the LPF at the posterolateral angle of the prostate on the posterior side of the NVB, as either clear, unclear, or absent. When leaves connecting the DF and LPF were unclear or absent, the mesorectal loose tissue appeared continuous with the NVB. Periprostatic nerves were not restricted at the posterolateral corner of the prostate, but their distribution extended medio-posteriorly in the DF [7,14]. These findings might be useful for understanding that the NVB is not restricted and that colorectal surgeons should pay careful attention to the anterior dissection plane of the rectum during total mesorectal excision.

Our study showed that all or most DF leaves were fused with the prostatic capsule near the base of the seminal vesicles. Notably, when the fascial space between the prostate and rectum was narrower without interposed mesorectal loose tissues at or near the midsagittal line, all or most DF leaves were fused with the prostatic capsule. Therefore, the dissection plane between the prostatic capsule and DF does not exist at the fusion site, because of the fascial complex between DF leaves and the prostatic capsule. Subsequently, periprostatic nerves were embedded in the combined capsule in a small area. Villers et al. [5] reported that muscular bundles and collagenous fibers of DF behind the vas deferens blend with central zone stroma and the ejaculatory duct sheath at the junction of the base of the prostate with the seminal vesicles and vas deferens. Kiyoshima et al. [6] reported that, in 79 surgically obtained specimens, DF was fused with the prostatic capsule at the center of the prostatic posterior aspect in 97% of cases. Hisasue et al. [15] reported the distribution of neuronal nitric oxide synthase positive nerve fibers, which are candidates for parasympathetic pro-erectile nerves [16]. They found that the distribution of these nerve fibers at 5- to 6-o’clock positions on the base of the prostate was 13.4% in the same hemisphere slice around the prostate in 23 specimens from non-nerve-sparing radical prostatectomy. However, what these nerves that are embedded in the fusion site innervate remains unclear. During RARP, DF is recognized as a membranous structure between the prostate and rectum by tension when pulling the seminal vesicles, vas deferens, and prostate ventrally. Simultaneously, the fusion site with DF and the prostatic capsule near the seminal vesicle-prostate junction are visible. DF between the seminal vesicles and rectum should first be cut at the midline to avoid entering the prostatic capsule. After this incision, a mesh-like structure behind the posterior aspect of the prostate corresponds with loose connective tissue and multiple leaves, and a flexible approach to the apex is easily found [17,18]. In cases of intra- or interfascial dissection, the dissection plane should be as close to the prostate as possible to avoid iatrogenic positive margins. Therefore, double cutting of DF while avoiding the fusion site is necessary to perform nerve-sparing procedures [19]. In cases of extrafascial dissection, the dissection plane unfolds at the loose connective tissue of DF to the apex. If advanced cancer is present at the border of the posterior aspect of the prostate, the dissection plane should be as close as possible to the rectal wall. This is because the fascial space between the prostate and rectum is not interposed with mesorectal loose tissues in some cases (Figure 5B) [17].

Distal to the fusion site, some DF leaves fuse with the prostatic capsule and/or the fascia propria of the rectum. DF reportedly conglutinates to the prostatic capsule with considerable frequency [4,5], whereas conglutination to the fascia propria of the rectum has not yet been reported, possibly because of difficulty in identification of the latter. During RARP, surgeons should be careful to avoid rectal injury during pre-rectal dissection because leaves of DF sometimes conglutinate to the fascia propria of the rectum.

DF converges on the grossly and histologically demonstrable posterior median raphe of rhabdosphincter and fibers of the RUM extended anteriorly into this fibrous raphe or central tendon of the perineum [20]. Soga et al. [21] reported that DF ends at the rhabdosphincter and the apical portion of the RUM. In our study, at least three specimens were found in which parts of the DF ended at the RUM. Of course, interindividual variations exist in the rhabdosphincter and RUM, and the shape of the rhabdosphincter is not always circular, but can show an omega shape [22]. In older men, the posterior rhabdosphincter is thin or absent in most cases. We suggest that the type of termination of DF depends on the size and shape of the rhabdosphincter and RUM [3].

Some potential limitations to this study should be considered when interpreting our findings. Variations of DF, such as the area in square millimeters and the numbers of leaves, were not evaluated quantitatively, because semiserial sections were used. Similarly, neither prostate size nor volume was evaluated before preparation of specimens for histological examination. Furthermore, because specimens including the rectum were difficult to obtain from young cadavers, no controls for likely changes with age were available.

## Conclusions

Site-dependent and interindividual variations in DF were observed in donated elderly male cadavers. All or most DF is fused with the prostatic capsule near the base of the seminal vesicles and some DF is fused with the fascia propria of the rectum posterior to the apex of prostate. Based on our results, surgeons should be aware of variations and search for them to create a suitable dissection plane to avoid iatrogenic positive margins and rectal injury.

## Abbreviations

DF: Denonvilliers’ fascia
HE: Hematoxylin and eosin staining
LPF: Lateral pelvic fascia
NVB: Neurovascular bundle
RARP: Robot-assisted radical prostatectomy
RUM: Rectourethralis muscle
